# Editorial: Adipose Tissue in the Cardiovascular Homeostasis and Disease

**DOI:** 10.3389/fphar.2021.803199

**Published:** 2021-11-24

**Authors:** Joshua T. Butcher, Ana P. Davel, Thiago Bruder-Nascimento

**Affiliations:** ^1^ Department of Physiological Sciences, College of Veterinary Medicine, Oklahoma State University, Stillwater, OK, United States; ^2^ Department of Structural and Functional Biology, Institute of Biology, University of Campinas–UNICAMP, Campinas, Brazil; ^3^ Department of Pediatrics, Center for Pediatrics Research in Obesity and Metabolism (CPROM), Vascular Medicine Institute (VMI), University of Pittsburgh, Pittsburgh, PA, United States

**Keywords:** adipose tissue, cardiovascular, adipokines, lipid, vascular

From an evolutionary perspective, adipocyte signaling would not have been a significant priority until the past 40 years, when the increased availability of a diet high in carbohydrates and fats (the Western Diet) dramatically pushed obesity-derived cardiovascular dysfunction to the forefront of health concerns. As the body’s largest endocrine organ, adipose tissue is predominantly composed of adipocytes as well as containing a small fraction of other cell types (ex: fibroblasts, vascular and immune cells). However, due to the broad dispersion of adipose tissue throughout the body and intra-organ location, its ability to contribute to and alter signaling cascades via adipokines magnify its importance in cardiovascular homeostasis. Indeed, adipose tissue plays a crucial role in health and disease, whether through expansion (obesity), or contraction (lipodystrophy) or dysfunction. This Research Topic was designed to highlight the multi-dimensional impact that adipose tissue has on systemic signaling throughout the body, with a focus on the cardiovascular system.

A highlight of the work enclosed in this Research Topic is the comprehensive models used to focus on the crosstalk of adipose and cardiovascular function, including human coronary arterioles, meta-analysis, and rodent models. A direct examination in humans of how epicardial adipose tissue (EAT) is altered in coronary artery disease (CAD) was shown by Wang et al. They determined that EAT in CAD patients possessed altered/dysfunctional transcriptomes and increased adipocyte size, likely contributing to the overall progression and development of CAD. Differentially expressed genes were used to construct a protein-protein interaction network and hub genes identified and validated, revealing a crucial interaction between inflammatory cells and chemokine signaling. Stephen T. O’Rourke’s group explored the effect of apelin on BK_Ca_ channels in the coronary vasculature. Importantly, using patch clamping and wire myography, they showed that apelin does not inhibit BK_Ca_ currents or nitric oxide-induced relaxation, an effect that contrasts with cerebral vasculature (Mughal et al.). Han et al. performed a meta-analysis focused on to establish the effectiveness of beta-blockers in preventing cardiac events in long QT syndrome patients according to gender, age, and QTc interval.

Importantly, work enclosed in this Research Topic also highlighted compounds that could be used to alter advanced disease states, like atherosclerosis. *Astragalus membranaceus* is a low cost medicinal herb normally used in several herbal formulations in the practice of traditional Chinese medicine and major components include polysaccharides, saponins, and flavone. In a clinically relevant study led by Dr. Fan’s group, the authors investigated the effects of total flavone of *Astragalus membranaceus* (TFA) treatment on atherosclerosis and hepatic steatosis in ApoE deficient mouse (ApoE KO, a well-established mouse model to study atherosclerosis). The authors observed that long-term TFA supplementation attenuates atherosclerosis development in high fat diet (HFD) treated ApoE KO mice, which was associated with a striking improvement of lipid disorder and hepatic outcomes (Ma et al.). In addition, TFA reduces lipid retention and uptake, as well as increases lipid efflux in isolated macrophages. Taken together, this study shows that TFA supplementation has multiple beneficial effects on cardiovascular readout by regulating lipid metabolism, hepatic outcomes, and by modulating macrophages and endothelial cells directly.

Pivoting away from the negative role of adipocyte tissue, Liu et al., examined the role of adiponectin in protecting against cardiomyocyte apoptosis, which plays a role in the development and progression of heart failure. Adiponectin can be found in the heart and skeletal muscle, but it is best known and explored in adipocytes, therefore is recognized as an adipokine (molecule produced by adipose tissue). In this interesting manuscript, the authors observed that LPS treatment induced immune cells infiltration and myocardial cell necrosis, which was abrogated by pre-treating the mice with adiponectin. Such changes were associated with a regulation of connexin 43 expression, an abundant cardiac protein that plays major role on cardiomyocyte apoptosis. To understand the signaling pathways by which adiponectin confers cardiac protection in LPS-treated mice, rat cardiomyocytes were treated with LPS in presence of adiponectin and the authors found that adiponectin blocks LPS-induced apoptosis via regulating PI3K/AKT pathways. This set of data has many clinical implications by demonstrating the positive effects of adiponectin on controlling sepsis severity, but also it has valuable implications in understanding why obese subjects (with lower levels of circulating adiponectin) present with a larger risk of death in sepsis conditions.

This Research Topic also contains reviews that highlight the distinctive role that perivascular adipose tissue (PVAT) can play, not only through diverse adipocytes (brown, white, beige), but also in its secretome and anatomical distribution (Li et al.). Further reviews focus on specific microenvironments, such as the prostate (Passos et al.) and atherosclerosis (Liu et al.) and how targeting PVAT via intermittent fasting can reduce inflammation and drive remodeling of adipocyte tissues (Dwaib et al.). Uniquely, a review by Dr. Jennifer Thompson’s group drew attention to the role of plasticizers, which certainly play a ubiquitous role in our society today, but yet have a poorly defined role as endocrine disruptors (Callaghan et al.). The pervasive role of these compounds, whether it is bisphenols (the most commonly known is BPA) or phthalates, is highlighted in the review, along with their impacts on adipogenesis, adipokine production, and inflammation. The protective role of PVAT is also impacted in the setting of obesity. Victorio et al. investigated whether the effect of obesogenic diets on PVAT function is dependent on sex. The authors found that PVAT dysfunction in response to two obesogenic HFD occurs early in females compared to age-matched males, suggesting a susceptibility of the female sex to obesity-induced PVAT dysfunction. The data illustrate the importance of the duration and composition of obesogenic diets for investigating sex-specific treatments and pharmacological targets for obesity-induced vascular complications.

Taken together, the enclosed work advances and clarifies our understanding of the cross-talk between adipose tissue and the cardiovascular system. This research informs on the foundational understanding of adipose tissue and its role in cardiovascular homeostasis, highlights new directions, and hopefully guides therapeutic targets in pathophysiological conditions driven by lipid disorders.

